# Tumor-microenvironment-mediated immune escape drives trastuzumab resistance in HER2-positive gastric cancer

**DOI:** 10.1093/gastro/goag068

**Published:** 2026-07-31

**Authors:** Huifang Lv, Wanying Zhao, Sai-Qi Wang, Yunduan He, Caiyun Nie, Xiao-Bing Chen

**Affiliations:** Department of Oncology, The Affiliated Cancer Hospital of Zhengzhou University & Henan Cancer Hospital, Henan Province Engineering Research Center for of Intractable Digestive Tract Tumor Precision Therapy, Henan Province Engineering Technology Research Center for Digestive Tract Tumor Precision Therapy, Zhengzhou Key Laboratory of Precision Therapy of Gastrointestinal Cancer, Zhengzhou 450008, Henan, P. R. China; State Key Laboratory of Metabolic Dysregulation & Prevention and Treatment of Esophageal Cancer, Zhengzhou University, Zhengzhou 450052, Henan, P. R. China; Cancer Hospital, The First Affiliated Hospital (College of Clinical Medicine), Henan University of Science and Technology, Luoyang 471003, Henan, P. R. China; Department of Oncology, The Affiliated Cancer Hospital of Zhengzhou University & Henan Cancer Hospital, Henan Province Engineering Research Center for of Intractable Digestive Tract Tumor Precision Therapy, Henan Province Engineering Technology Research Center for Digestive Tract Tumor Precision Therapy, Zhengzhou Key Laboratory of Precision Therapy of Gastrointestinal Cancer, Zhengzhou 450008, Henan, P. R. China; State Key Laboratory of Metabolic Dysregulation & Prevention and Treatment of Esophageal Cancer, Zhengzhou University, Zhengzhou 450052, Henan, P. R. China; Department of Oncology, The Affiliated Cancer Hospital of Zhengzhou University & Henan Cancer Hospital, Henan Province Engineering Research Center for of Intractable Digestive Tract Tumor Precision Therapy, Henan Province Engineering Technology Research Center for Digestive Tract Tumor Precision Therapy, Zhengzhou Key Laboratory of Precision Therapy of Gastrointestinal Cancer, Zhengzhou 450008, Henan, P. R. China; State Key Laboratory of Metabolic Dysregulation & Prevention and Treatment of Esophageal Cancer, Zhengzhou University, Zhengzhou 450052, Henan, P. R. China; Department of Oncology, The Affiliated Cancer Hospital of Zhengzhou University & Henan Cancer Hospital, Henan Province Engineering Research Center for of Intractable Digestive Tract Tumor Precision Therapy, Henan Province Engineering Technology Research Center for Digestive Tract Tumor Precision Therapy, Zhengzhou Key Laboratory of Precision Therapy of Gastrointestinal Cancer, Zhengzhou 450008, Henan, P. R. China; State Key Laboratory of Metabolic Dysregulation & Prevention and Treatment of Esophageal Cancer, Zhengzhou University, Zhengzhou 450052, Henan, P. R. China; Department of Oncology, The Affiliated Cancer Hospital of Zhengzhou University & Henan Cancer Hospital, Henan Province Engineering Research Center for of Intractable Digestive Tract Tumor Precision Therapy, Henan Province Engineering Technology Research Center for Digestive Tract Tumor Precision Therapy, Zhengzhou Key Laboratory of Precision Therapy of Gastrointestinal Cancer, Zhengzhou 450008, Henan, P. R. China; State Key Laboratory of Metabolic Dysregulation & Prevention and Treatment of Esophageal Cancer, Zhengzhou University, Zhengzhou 450052, Henan, P. R. China; Cancer Hospital, The First Affiliated Hospital (College of Clinical Medicine), Henan University of Science and Technology, Luoyang 471003, Henan, P. R. China

**Keywords:** gastric cancer, HER2, trastuzumab, drug resistance

## Abstract

The combination of trastuzumab and chemotherapy, with or without pembrolizumab, is the current first-line standard of care for patients with human epidermal growth factor receptor 2 (HER2)-positive advanced gastric cancer and gastroesophageal junction cancer. However, upon disease progression, subsequent therapies often yield limited clinical benefit. The mechanisms of drug resistance to trastuzumab remain unresolved. Current studies on its resistance mechanisms have largely focused on alterations in the HER2 pathway, including HER2 heterogeneity, reduced or absent HER2 expression, variations in HER2 dimerization, and mutations in downstream components, among others. Crucially, as a monoclonal antibody, the antitumor activity of trastuzumab is partially mediated by the immune system, yet the immunology-related mechanisms of resistance are frequently overlooked. In this review, we systematically analyse the outcomes of both successful and failed clinical trials of anti-HER2 agents to propose that immune escape within the tumor microenvironment is a key driver of trastuzumab resistance in HER2-positive gastric cancer.

## Introduction

Gastric cancer (GC) is one of the most common digestive tumors in China. According to the 2022 GLOBOCAN data, GC ranks fifth in both incidence and mortality globally [1]. In China, the disease is particularly prevalent, accounting for 43.9% of new cases worldwide and ranking third in cancer incidence and fifth in mortality [2]. Approximately half of patients are diagnosed at an advanced stage, contributing to a poor prognosis and a low 5-year survival rate [3].

Human epidermal growth factor receptor 2 (HER2) belongs to the HER family and can be expressed on the surface of cancer cells. HER2 directly activates downstream signaling pathways, including the RAS–MAPK and PI3K–AKT pathways, thereby promoting cancer-cell proliferation and migration [1, 2]. Approximately 10.4%–34% of GC patients are HER2-positive (IHC HER2 3+ or HER2 2+/ISH+) [4–8]. In the pre-trastuzumab era, patients with HER2-positive GC had a poorer prognosis and the duration of disease control with standard chemotherapy was no more than 6 months [9].

### Current status of first-line treatment for HER2-positive GC

Trastuzumab, a recombinant humanized anti-HER2 monoclonal antibody, can bind to the HER2 on cancer cells, block HER2 dimerization, and exert its antitumor effects [10, 11]. The ToGA trial was a large-scale, randomized Phase III study that demonstrated significant benefits of trastuzumab combined with chemotherapy (cisplatin and fluoropyrimidine). Patients receiving this regimen had significantly longer median overall survival (mOS; 13.8 vs 11.1 months), longer median progression-free survival (mPFS; 6.7 vs 5.5 months), and a higher objective response rate (ORR; 47.3% vs 34.5%) and disease control rate (DCR; 78.9% vs 69.3%) than those receiving chemotherapy alone [12]. These results established a new standard for the first-line treatment of HER2-positive advanced GC and gastroesophageal junction (GEJ) cancer, marking the era of targeted therapy for such patients. Subsequent studies, such as herMES, HERBIS-1, CGOG1001 [13–18], and EVIDENCE [19], explored different chemotherapy backbones combined with trastuzumab, further confirming its therapeutic role. Despite this, most patients eventually develop resistance.

PD-1 inhibitors, such as nivolumab, can recognize and bind to PD-L1 on the surface of cancer cells, relieve T-cell inhibition, and enhance T-cell–induced immune activity [20, 21]. However, retrospective analysis of the efficacy of nivolumab in the third-line or later treatment of HER2-positive advanced GC showed an ORR of only 6%, indicating that patients with HER2-positive GC do not benefit significantly from immune checkpoint inhibitors (ICIs) alone in the later-line treatment [21]. Combining PD-1 monoclonal antibodies with trastuzumab and chemotherapy has shown promise in improving efficacy in first-line treatments. A Phase II study conducted by Memorial Sloan Kettering Cancer Center evaluated the triple regimen of pembrolizumab, trastuzumab, and chemotherapy, and demonstrated encouraging results, with an mOS of 27.3 months and an ORR as high as 91% [22]. The PANTHERA trial (NCT02901301) further evaluated this combination as a first-line treatment and reported an ORR of 76.7%, a DCR of 97%, and an mOS 19.3 months [23]. The KEYNOTE-811 trial—a randomized, double-blind Phase III study—evaluated the efficacy of pembrolizumab plus trastuzumab and chemotherapy compared with placebo plus trastuzumab and chemotherapy as first-line treatment for HER2-positive unresectable or metastatic GC or GEJ cancer and the first interim analysis showed an ORR of 74.4% in the pembrolizumab group, with a 22.7% improvement in ORR compared with the placebo group, and an increase in the complete response rate from 3% to 11% [24]; the third interim analysis showed longer progression-free survival (PFS) (10.0 vs 8.1 months) [25]. It will provide a new treatment strategy for first-line treatment in HER2-positive advanced GC. The final analysis confirmed that adding pembrolizumab to trastuzumab and chemotherapy significantly improved PFS and overall survival (OS). The survival benefit from adding pembrolizumab to the regimen was primarily driven by patients with PD-L1, with a combined positive score (CPS) of ≥1. In 2021, the US Food and Drug Administration (FDA) granted accelerated approval for pembrolizumab in combination with trastuzumab and chemotherapy for the first-line treatment of HER2-positive advanced GC or GEJ cancer, based on the ORR in the overall study population and regardless of the PD-L1 expression. Following the availability of final OS data, the FDA converted this indication into regular approval in 2025. This regular approval explicitly limits the indication to adult patients whose tumors express PD-L1 with a CPS of ≥1. The INTEGA study investigated the possibility of chemo-free regimens [26]. This randomized Phase II trial evaluated the efficacy of trastuzumab plus nivolumab combined with either FOLFOX or ipilimumab in patients with HER2-positive advanced GC. The FOLFOX group showed superior efficacy, with an mOS of 21.8 months and a 1-year OS rate of 70% [27]. In contrast, the OS of the ipilimumab group did not improve, achieving a median OS of only 16.4 months and a 1-year OS rate of 57% [26]. Therefore, FOLFOX may be more suitable for patients requiring rapid tumor regression or those with a high tumor burden. Conversely, ipilimumab might be considered for patients who are chemotherapy-intolerant or in whom long-term immune activation is desired, although its higher immunotoxicity risk requires careful consideration.

Novel drugs, such as zanidatamab (ZW25) and anbenitamab (KN-026), are anti-HER2 bispecific antibodies. These agents can simultaneously bind to two distinct non-overlapping epitopes of HER2, similar to the binding sites targeted by trastuzumab and pertuzumab, thereby exerting their antitumor effects through dual blockade of the HER2 signaling pathway [28–30]. ZW25 in combination with chemotherapy was initially explored as a salvage treatment for HER2-positive advanced GC and demonstrated promising efficacy. Subsequently, the combination of ZW25 with tislelizumab and chemotherapy was evaluated as a first-line treatment for HER2-positive GC/GEJ cancer. The results revealed an ORR of 72.7% and a DCR of 100%. The HERIZON-GEA-01, a Phase III trial, evaluated zanidatamab plus chemotherapy with or without tislelizumab versus trastuzumab plus chemotherapy in patients with previously untreated HER2-positive advanced gastroesophageal adenocarcinoma [31]. It showed significant PFS improvement in the zanidatamab-plus-chemotherapy group (mPFS 12.4 vs 8.1 months, hazard ratio [HR] = 0.65, *P *< 0.001). The OS was also significantly improved in the zanidatamab-plus-tislelizumab-plus-chemotherapy group (mOS 26.4 vs 19.2 months, HR = 0.72, *P *= 0.004). It showed an ORR of ∼70% in the zanidatamab group.

**Figure 1.**
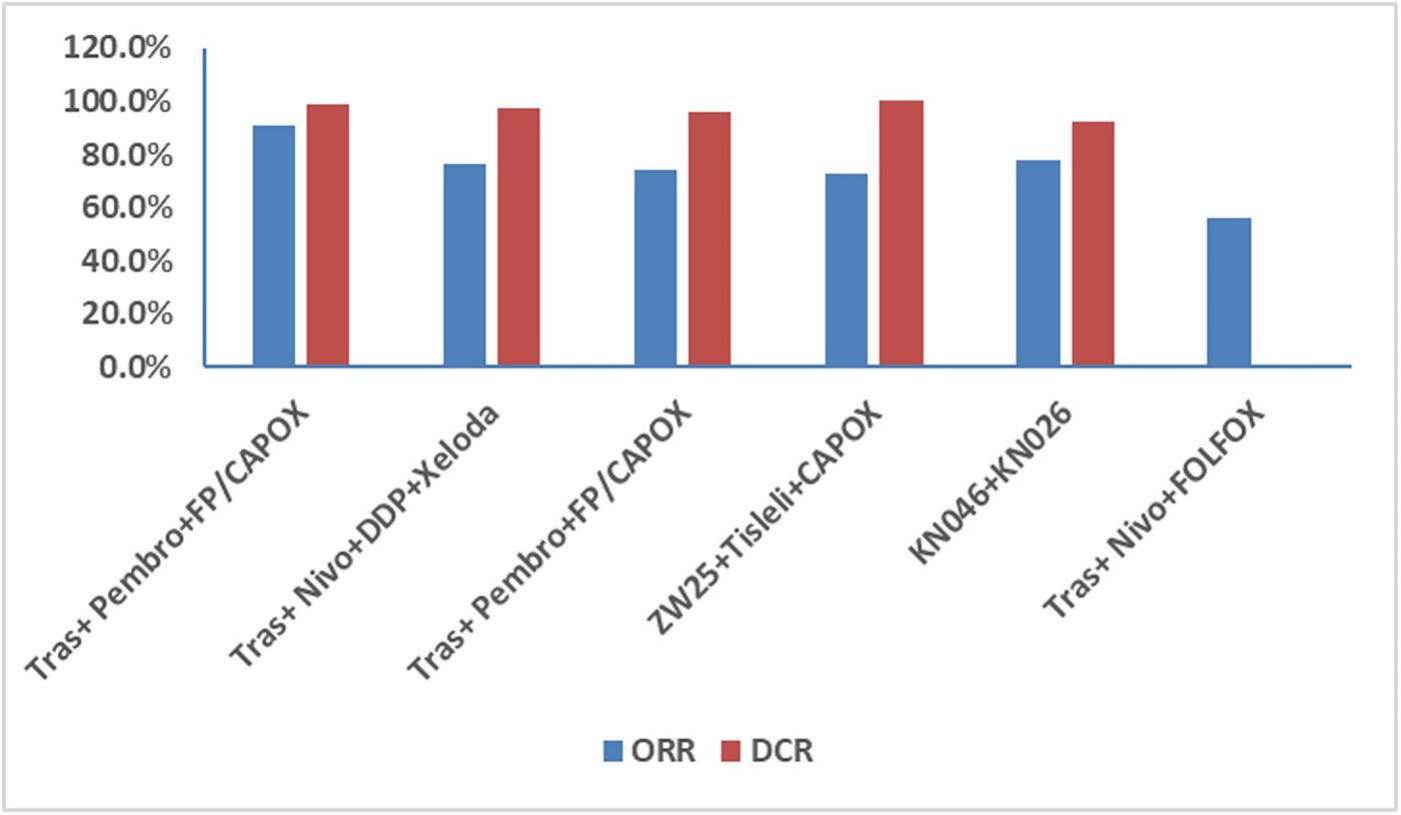
ORR and DCR in different clinical studies.

The efficacy of KN026 in HER2-positive GC has been evaluated across multiple clinical settings. The Phase III KC-WISE trial assessed KN026 monotherapy as a second-line treatment for advanced HER2-positive GC, demonstrating an ORR of 56% [32]. Building on these findings, the combination of KN026 with KN046, an investigational immunotherapy agent targeting both the PD-L1 and CTLA-4 pathways, has been evaluated as a first-line treatment for locally advanced unresectable or metastatic HER2-positive GC or GEJ cancer. This combination regimen yielded substantial improvements in clinical outcomes, with an ORR of 77.8% and a DCR of 92.6% [33]. Collectively, these findings suggest that combining anti-HER2 agents with immunotherapy represents a promising strategy to enhance the response rates in patients with HER2-positive GC (Fig. 1).

### Dilemma of second-line-and-above treatment for HER2-positive GC

Despite the success of first-line trastuzumab-based therapy, treatment options after progression remain limited. For years, chemotherapy with or without anti-angiogenic agents was the mainstay of the second-line treatment of HER2-positive GC [34–38] but most trials failed to show significant benefit. Despite many trials being explored, most failed. This outcome changed until the advent of antibody-drug-conjugates (ADCs) and anti-HER2 bispecific antibodies. KC-WISE [32], a Phase III trial, aimed to evaluate the efficacy and safety of anbenitamab plus chemotherapy compared with placebo plus chemotherapy in patients with HER2-positive advanced GC or GEJ adenocarcinoma who had failed previous first-line trastuzumab-containing therapy. It demonstrated striking efficacy for anbenitamab plus chemotherapy: median PFS, 7.1 vs 2.7 months (HR = 0.25, *P *< 0.001); median OS, 19.6 vs 11.5 months (HR = 0.29, *P *< 0.001); ORR, 55.8% vs 10.8%.

ADC drugs have a dual effect of targeted therapy and cytotoxic killing. Trastuzumab deruxtecan (T-DXd, DS8201) has demonstrated significant antitumor efficacy and controllable safety in multistage and multi-regional clinical studies of HER2-positive advanced GC. DESTINY-Gastric01, a Phase II clinical trial conducted in Japan and South Korea (*n *= 187), compared the efficacy of T-DXd with chemotherapy in the later-line treatment of advanced HER2-positive GC [39]. The results showed that the ORR in the T-DXd group reached 51%, which was significantly higher than the 14% achieved in the chemotherapy group; the mOS was 12.5 vs 8.4 months (HR = 0.59) and the mPFS was 5.6 vs 3.5 months (HR = 0.47). DESTINY-Gastric02, a Phase II confirmatory trial in Western populations (*n *= 79), further confirmed the efficacy of T-DXd in second-line treatment, with an ORR of 42%, an mPFS of 5.6 months, and an mOS of 12.1 months [40]. DESTINY-Gastric03, a Phase Ib/II trial, explored the application of T-DXd ± chemotherapy ± pembrolizumab in first-line treatment [41]. In Part 2, the regimen of T-DXd combined with fluoropyrimidine-based chemotherapy and pembrolizumab achieved an ORR of 58% and the ORR was as high as 70% in the population with a PD-L1 CPS of ≥1. The global Phase III clinical trial DESTINY-Gastric04 (*n *= 490) compared the efficacy of T-DXd with ramucirumab plus paclitaxel in second-line treatment [42]. The results showed that the T-DXd group had significantly improved OS (14.7 vs 11.4 months, HR = 0.70, *P *< 0.001), prolonged PFS to 6.7 months (4.3 months in the control group, HR = 0.58), and increased ORR to 44.3% (20.2% in the control group). In the DESTINY-Gastric06 (*n *= 95), the ORR for the T-DXd treatment was 28.8%, the median PFS was 5.7 months, the median OS was 11.1 months, and the incidence of ILD was only 3.2%, all of which were grade 1–2, showing more favorable safety [43].

For another HER2-targeted ADC, the Phase I study of disitamab vedotin (RC48) showed that, in a population of HER2-positive advanced solid tumors including 24 GC patients, the ORR of the GC patients at the recommended Phase II dose was 21.1% [44]. Its pivotal Phase II trial RC48-C008 confirmed an ORR of 23.6%, an mPFS of 4.1 months, and an mOS of 7.5 months in patients with pretreated HER2-overexpressing advanced GC.

We summarized the key trials of anti-HER2 agents in GC (Table 1) and found that DS-8201a [39] and RC48-ADC [45] are effective in treating HER2-positive GC whereas T-DM1 [46] is not. This indicates that their antitumor effect mainly depends on direct killing by cytotoxic drugs. Firstly, ADC drugs are composed of anti-HER2 antibodies, linkers, and cytotoxic drugs. DS-8201a and RC48-ADC feature cleavable linkers that, upon internalization, release their cytotoxic payloads, thereby enabling a bystander effect. This released payload can kill adjacent cancer cells, irrespective of their HER2-expression status—a phenomenon known as the bystander effect. Beyond eliminating HER2-low or HER2-negative neighboring tumor cells, the bystander effect may also impact the tumor microenvironment (TME) by releasing antigens that stimulate adaptive immune responses, potentially synergizing with ICIs. This indirect immune activation likely contributes to the superior efficacy of these ADCs compared with T-DM1, which lacks the bystander effect due to its non-cleavable linkers, thus limiting its tumor-killing ability. Moreover, the efficacy of ADC drugs is related to the types of coupled chemotherapeutic agents. The DXd in DS 8201a is a highly active topoisomerase inhibitor. Monomethyl-aurestatin E, which is coupled in RC48-ADC, is a microtubule polymerization inhibitor. Both DS 8201a and RC 48-ADC are used in the second-line treatment of GC. DM1, the maytansinoid payload conjugated to T-DM1, exerts its antitumor effects by binding to tubulin at the vinca alkaloid-binding site, thereby disrupting microtubule polymerization [47]. However, while preclinical studies have shown that DM1 exhibits some activity against GC cells, vinca alkaloids themselves are not used clinically for this disease.

**Table 1.** Summary of anti-HER2 drug trials in GC

Trial	Phase	Setting	Treatment	Efficacy
ToGA	III	First-line	Trastuzumab+ CT vs CT	OS: 13.8 vs 11.1 months (HR 0.74, 95% CI 0.60–0.91, *P *= 0.0046); PFS: 6.7 vs 5.5 months (HR 0.71, 95% CI 0.59–0.85, *P* = 0.0002); ORR: 47% vs 35% (*P *= 0.0017)
NCT02954536	II	First-line	Pembrolizumab + trastuzumab + CT	PFS: 13.0 months (95% CI 8.6–NR); OS: 27.3 months (95% CI 18.8–NR); ORR: 91%
KEYNOTE-811	III	First-line	Pembrolizumab + trastuzumab + CT vs placebo + trastuzumab + CT	OS: 20.2 vs 16.8 months (HR 0.80, 95% CI 0.67–0.94, *P *= 0.004); PFS: 10.0 vs 8.1 months (HR = 0.73, 95% CI 0.61–0.87)
JACOB	III	First-line	Pertuzumab + trastuzumab + CT vs placebo + trastuzumab + CT	OS: 17.5 vs 14.2 months (HR 0.84, 95% CI 0.71–1.00, *P *= 0.057); PFS: 8.5 vs 7.0 months (HR 0.73, 95% CI 0.62–0.86, *P *= 0.0001); ORR: 56.7% vs 48.3% (*P *= 0.026)
LOGiC	III	First-line	Lapatinib + CT vs CT	OS: 12.2 vs 10.5 months (HR 0.91, 95% CI 0.73–1.12, *P *= 0.32); PFS: 6.0 vs 5.4 months (HR 0.82, 95% CI 0.68–1.00, *P *= 0.038); ORR: 53% vs 39% (*P *= 0.0031)
T-ACT	II	Second-line	Trastuzumab + CT vs CT	PFS: 3.7 vs 3.2 months (HR 0.91, 80% CI 0.67–1.22, *P *= 0.33); OS: 10.2 vs 10.0 months (HR 1.2, 95% CI 0.75–2.0, *P *= 0.20); ORR: 33% vs 32% (*P *= 1.00)
TyTAN	III	Second-line	Lapatinib + CT vs CT	OS: 11.0 vs 8.9 months (HR 0.84, 95% CI 0.64–1.11, *P *= 0.10); PFS: 5.4 vs 4.4 months (HR 0.85, 95% CI 0.63–1.13, *P *= 0.24); ORR: 27% vs 9% (*P *< 0.001)
GATSBY	II/III	Second-line	Trastuzumab emtansine vs CT	OS: 7.9 vs 8.6 months (HR 1.15, 95% CI 0.87–1.51, *P *= 0.86)
CP-MGAH22–05	Ib/II	Second-line	Margetuximab + pembrolizumab	OS: 12.48 months (95% CI 9.07–14.09); PFS: 2.73 months (95% CI 1.61–4.34); ORR: 18.48% (95% CI 11.15–27.93)
DESTINY-Gastric01	II	Third-line or later	Trastuzumab deruxtecan vs CT	PFS: 5.6 vs 3.5 months (HR 0.47, 95% CI 0.31–0.71, *P *= 0.0003); OS: 12.5 vs 8.4 months (HR 0.59, 95% CI 0.39–0.88, *P *= 0.0097); ORR: 42.9% vs 12.5%
ASPEN-01	I	Second-line	Trastuzumab + evorpacept vs trastuzumab	ORR: 21.1% (vs control not applicable)
HELOISE	IIIb	First-line	SoC trastuzumab + CT vs HD trastuzumab + CT	mOS: 12.5 vs 10.6 months (HR 1.24, 95% CI 0.86–1.78, *P *= 0.2401)
HER-RAM	Ib/II	Second-line	CT + trastuzumab + ramucirumab	PFS: 7.1 months (95% CI 4.8–9.4); OS: 13.6 months (95% CI 9.4–17.7); ORR: 54% (27/50); DCR: 96% (48/50)
DESTINY-Gastric01	II	Third-line or later	Trastuzumab deruxtecan vs CT	OS: 12.5 vs 8.4 months (HR 0.59, 95% CI 0.39–0.88, *P *= 0.01); PFS: 5.6 vs 3.5 months (HR 0.47, 95% CI 0.31–0.71); ORR: 51% vs 14%
DESTINY-Gastric02	II	Second-line	Trastuzumab deruxtecan	OS: 12.1 (95% CI 9.0–14.3); PFS: 5.6 months (95% CI 4.2–8.3); ORR: 42% (95% CI 30.8–53.4)
DESTINY-Gastric03	Ib/II	First-line	Trastuzumab deruxtecan alone or in combination with chemotherapy (such as fluoropyrimidine-based regimens) or immunotherapy (such as pembrolizumab), etc.	Not yet fully reported
DESTINY-Gastric04	III	Second-line	Trastuzumab deruxtecan vs ramucirumab + paclitaxel	OS: 14.7 vs 11.4 months (HR 0.7, 95% CI 0.55–0.90, *P *= 0.0044); PFS: 6.7 vs 4.3 months (HR 0.74, 95% CI 0.59–0.92, *P *= 0.0074); ORR: 44.3% vs 20.2% (*P *< 0.001)
DESTINY-Gastric06	II	Third-line or later	Trastuzumab deruxtecan	OS: 11.1 months (95% CI 7.7–13.7); PFS: 5.7 months (95% CI 4.0–6.8); ORR: 28.8% (95% CI 18.8–40.6)
RC48-C008	II	Third-line or later	Disitamab vedotin (RC48)	OS: 7.5 months (95% CI 6.7–9.9); PFS: 4.1 months (95% CI 3.7–4.9); ORR: 23.6%

CT = chemotherapy, CI = confidence interval, SoC = standard-of-care, HD = higher-dose.

In addition, Xu *et al.* [48] reported the first-in-human Phase I trial of TQB2102—a novel HER2 bispecific ADC targeting ECD2 and ECD4 with a topoisomerase I inhibitor payload. In 10 patients with evaluable HER2-positive GC, the ORR was 70% (7 PR), the DCR was 90%, and the 6-month PFS rate was 90%. These preliminary data suggest that TQB2102 may offer high efficacy with a favorable safety profile, warranting further investigation.

### Improvement in the TME enhances the effect of trastuzumab in HER2-positive GC

Given the limited success of second-line therapies, recent attention has shifted toward understanding the way in which the TME modulates the efficacy of trastuzumab. Emerging evidence has suggested that enhancing the immune landscape within the TME can potentiate the antitumor effects of trastuzumab. There are various anti-HER2 drugs used in the treatment of GC. Trastuzumab primarily exerts its effects through two mechanisms: blocking signaling pathways and inducing Fc-mediated immune responses [49]. It binds to the extracellular domain IV of HER2, forming HER2 homologous dimers and blocking downstream signaling pathway activation. This results in a classic receptor inhibitory effect.

Macrophages with an Fc gamma receptor (FcγR) can bind to trastuzumab and activate antibody-dependent cellular phagocytosis (ADCP), leading to tumor-cell elimination [50–53]. Additionally, trastuzumab binds to Fcγ receptors on natural killer cells, initiating antibody-dependent cellular cytotoxicity (ADCC). Recent studies have suggested that HER2 can inhibit the immune response by blocking the cGAS–STING pathway, further enhancing its immunomodulatory effects [54, 55]. Tumor-infiltrating lymphocytes (TILs) are recognized as prognostic biomarkers for trastuzumab [56]. Two different doses of trastuzumab combined with chemotherapy were investigated in patients with HER2-positive GC; the study found that the lower dose resulted in an mOS of 12.5 months, while the higher dose showed no significant improvement [57]. This suggests that the efficacy of trastuzumab may depend more on its immune-mediated effects rather than its blockage of the signaling pathway.

Lapatinib is a small-molecule tyrosine kinase inhibitor (TKI) that blocks HER-family dimerization, inhibits kinase domain phosphorylation, and prevents downstream signaling pathway activation, ultimately suppressing cancer-cell growth [58–60]. The LOGiC study investigated the effects of lapatinib combined with chemotherapy versus chemotherapy alone in patients with HER2-amplified gastric and gastroesophageal adenocarcinoma [61]. The results demonstrated that, compared to the control group, the lapatinib group did not show statistically significant improvements in mOS (12.2 vs 10.5 months) or mPFS (6.0 vs 5.4 months). The failure of this trial may suggest that simply blocking the downstream pathways of HER2 is insufficient to enhance the efficacy of anti-HER2 agents.

Pertuzumab binds to the extracellular domain II of HER2, thereby preventing HER2 heterodimerization and inhibiting cancer-cell growth [62]. Preclinical studies have demonstrated that the combination of trastuzumab and pertuzumab, which blocks dual HER2 dimerizations, exhibits stronger antitumor effects *in vitro* [60, 63]. However, while this dual blockade showed promise *in vitro*, it did not translate into clinical benefits *in vivo*. The JACOB study evaluated the efficacy of trastuzumab plus pertuzumab combined with chemotherapy as first-line treatment for HER2-positive advanced GC [64]. The results revealed no significant differences between the two groups in terms of mOS (17.5 vs 14.2 months) or mPFS (8.5 vs 7.0 months). These findings indicate that adding pertuzumab to standard treatment does not improve survival outcomes. This again highlights that merely intensifying the blockade of HER2 downstream pathways is insufficient to enhance clinical benefits. In light of the positive results from HERIZON-GEA-01 with the biparatopic antibody zanidatamab, this apparent failure can be reinterpreted. HER2-positive disease is highly heterogeneous and simply adding two monoclonal antibodies (i.e. trastuzumab + pertuzumab) provides only incremental “quantitative” enhancement of the HER2 blockade. In contrast, a true “qualitative” leap requires a more potent agent such as zanidatamab, which induces HER2 clustering and internalization, achieving more complete pathway suppression. Thus, the negative outcome of the JACOB study underscores that dual monoclonal antibody blockade is insufficient to overcome HER2 heterogeneity, while biparatopic antibodies can achieve a clinical breakthrough.

In addition, it is widely believed that classical targeted drugs are generally not used in combination with ICIs, while combinations such as PD-1 inhibitors plus CTLA-4 inhibitors or anti-angiogenic drugs can be employed for cancer treatment. For example, the co-administration of TKIs with PD-1/PD-L1 inhibitors is not currently considered in the treatment of non–small cell lung cancer, regardless of whether the TKIs are first-, second-, or third-generation agents [65]. However, multiple studies have demonstrated that the addition of PD-1 inhibitors to trastuzumab-based chemotherapy regimens yields greater efficacy in the first-line treatment of HER2-positive GC. The KEYNOTE-811 trial confirmed that adding pembrolizumab to trastuzumab and chemotherapy significantly increased the ORR and improved survival outcomes in these patients. Furthermore, data indicated that Evorpacept (ALX148), a CD47 monoclonal antibody, combined with trastuzumab exhibited antitumor activity [66]. The ASPEN-01 study aimed to evaluate the efficacy of evorpacept (ALX148) in HER2-positive cancers [66]. Among 19 patients who had developed resistance to trastuzumab, treatment with CD47 inhibitors plus trastuzumab yielded promising results: 4 patients achieved deep tumor regression and 1 patient experienced stable disease, resulting in an ORR of 21.1%. The HER-RAM trial sought to assess the efficacy of trastuzumab combined with ramucirumab and paclitaxel in the second-line treatment of HER2-positive GC [67]. The results demonstrated that this regimen yielded a PFS of 7.4 months and an OS of 13.6 months, significantly improving patient outcomes and preliminarily showing synergistic effects between trastuzumab and anti-angiogenic agents. In our own case report [68], we described a HER2-positive GC patient with acquired resistance to trastuzumab. Following disease progression on trastuzumab therapy, the patient was treated with a combination of trastuzumab, apatinib, and camrelizumab, achieving a PFS of 16 months. These data underscore that modulating the TME can enhance the therapeutic efficacy of trastuzumab in HER2-positive GC.

Therefore, through a systematic analysis of successful and failed outcomes of anti-HER2 agents in GC (Table 2), it is evident that trastuzumab resistance is closely associated with the TME in HER2-positive GC. The addition of drugs or strategies that improve the tumor immune microenvironment (TIME) can enhance the effectiveness of trastuzumab-based therapies.

**Table 2.** Impact of anti-HER2 agents on the immune microenvironment in GC

Project	Key efficacy outcome	Experiment group	Control group	Impact on immune microenvironment	Finding
LOGiC (first-line)		CT + lapatinib	CT	No significant immune modulation observed	No substantial immune modulation observed in this setting
	OS (months)	10.5	12.2		
	PFS (months)	5.4	6		
TyTAN (second-line)		CT + lapatinib	CT	No significant immune modulation observed	Lack of IME impact may contribute to negative trial
	OS (months)	11	8.9		
JACOB (first-line)		CT + trastuzumab + pertuzumab	CT + trastuzumab	Enhanced ADCC effect	Dual HER2 blockade with pertuzumab may augment trastuzumab-mediated ADCC
	OS (months)	17.5	14.2		
	PFS (months)	8.5	7		
KEYNOTE-811 (first-line)		CT + trastuzumab + pembrolizumab	CT + trastuzumab	Activate T cells and enhance ADCC	PD-1 blockade enhances T-cell activation and ADCC, potentiating antitumor immunity
	ORR (%)	74.4%	51.9%		
ASPEN-01 (second-line)		Trastuzumab + ALX148	trastuzumab	ALX148 enhances the ADCP mediated by therapeutic antibodies	Evorpacept (CD47 blocker) promotes macrophage-mediated tumor-cell phagocytosis
	ORR (%)	21.1%	–		
HELOISE (first-line)		SoC trastuzumab + CT	HD trastuzumab + CT	No significant immune modulation observed	Higher trastuzumab dose did not alter immune outcomes or survival
	OS (months)	12.5	10.6		
HER-RAM (second-line)		CT + trastuzumab + ramucirumab	CT + trastuzumab	VEGF inhibition/normalized tumor vasculature	VEGF inhibition normalizes vasculature, alleviates hypoxia, and enhances immune infiltration
	OS (months)	13.6	–		
	PFS (months)	7.1	–		

CT = chemotherapy, CI = confidence interval, SoC = standard-of-care, HD = higher-dose, ALX148: evorpacept, a CD47 blocker.  —, not available.

The TIME in HER2-positive GC is characterized by the infiltration of various immune cells, including tumor-associated macrophages (TAMs), natural killer cells, and CD8^+^ T cells. TAMs can be polarized toward an M2-like phenotype, which promotes immunosuppression and correlates with a poor response to trastuzumab. Furthermore, the expression of immune checkpoint molecules such as PD-L1 and CD47 on tumor cells is dynamically regulated by interactions with the TME, leading to adaptive immune resistance. Understanding these complex dynamics is crucial for elucidating the mechanisms of trastuzumab resistance.

Recent translational studies have provided deep insights into the TIME of HER2-positive GC. Cen *et al.* [69] systematically reported that HER2-positive GC exhibits distinct immune features: CD8^+^ T-cell infiltration correlates with better prognosis, while Tregs and M2-type TAMs are associated with poor outcomes. HER2 signaling can downregulate MHC-I via PI3K/AKT, leading to immune evasion. PD-L1 expression correlates with the TIL density, providing rationale for ICI combinations. Lv *et al.* [4] confirmed that HER2 positivity is positively associated with PD-L1 expression and HER2^+^/PD-L1^+^ double-positive patients have the worst prognosis, indicating they may benefit the most from combined immunotherapy.

Kwon *et al.* [70] performed whole-exome sequencing and multiplex immunofluorescence and identified three immune subtypes: immune-enriched, immune-exhausted, and immune-desert. Chen *et al.* [71] further revealed the heterogeneity of PD-L1 expression: PD-L1 on tumor cells (TC) correlates negatively with CD8^+^ T-cell infiltration, while PD-L1 on immune cells is associated with tertiary lymphoid structures (TLS). A combined score incorporating both outperforms CPS alone in predicting the response to trastuzumab plus ICI. Jiang *et al.* [72] analysed paired pre- and post-trastuzumab + chemo samples from 30 patients by using single-cell RNA sequencing and multiplex immunofluorescence. Responders showed increased CD8^+^ T cells and DCs after treatment, with clonal T-cell expansion. An early (Week 3) CD8^+^ T-cell increase of >20% predicted longer PFS. Nonresponders had enrichment of SPP1^+^ TAMs and cancer-associated fibroblasts, creating an immunosuppressive barrier. Sheng *et al.* [73] from Patrick Tan’s group used spatial transcriptomics and multiplex IF on 35 matched baseline and resistant samples, revealing spatial resistance mechanisms. They identified three spatial immune architectures: immune-enriched (25%, best response), immune-excluded (45%, T cells confined to stroma), and immune-desert (30%, worst prognosis). Resistance mechanisms included physical barriers by activated fibroblasts, the formation of immunosuppressive niches with SPP1^+^ TAMs and Tregs colocalization, and the spatial heterogeneity of HER2 expression. Tumor-residing TLS correlated with better prognosis. A spatial risk score incorporating immune exclusion, SPP1^+^ TAM density, TLS maturity and location, and HER2 spatial heterogeneity effectively predicted the outcome (HR = 3.2).

These translational findings have important clinical implications: the development of immune subtype-based personalized therapy, early CD8^+^ T-cell dynamics as a predictive biomarker, targeting SPP1^+^ TAMs or physical barriers to overcome resistance, and the need for dynamic monitoring via liquid biopsy.

### The role of the TME in trastuzumab resistance in HER2-positive GC

**Figure 2.**
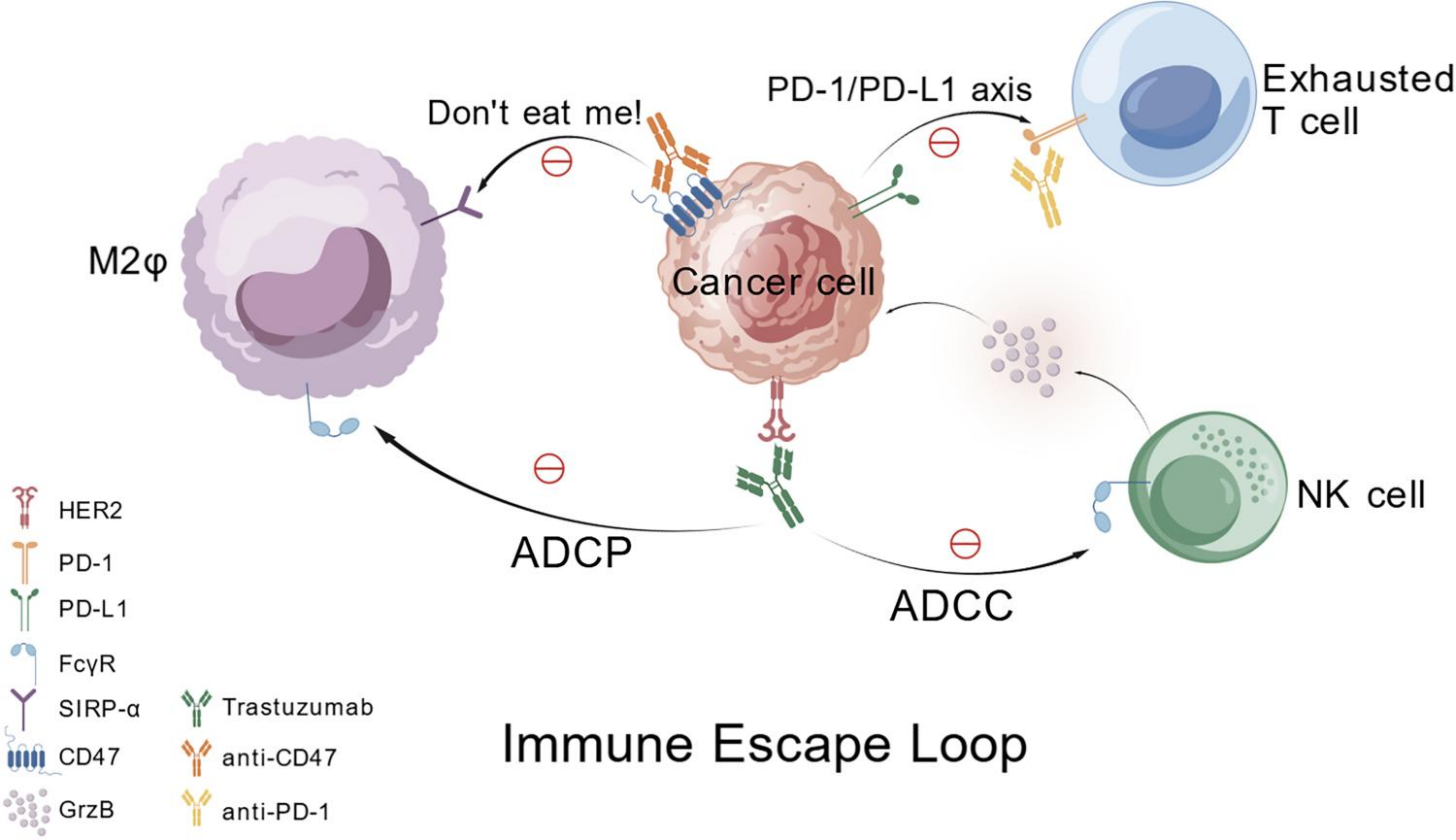
Immune mechanisms of trastuzumab resistance. M2φ = M2 macrophage, PD-1 = programmed cell death protein 1, PD-L1 = programmed death-ligand 1, NK cell = natural killer cell, SIRPα = signal regulatory protein alpha, CD47 = cluster of differentiation 47, GrzB = granzyme B.

Immune checkpoints PD-L1 and CD47 play a key role in mediating adaptive immune escape and innate immune suppression. Previous studies have shown that trastuzumab indirectly upregulates PD-L1 expression on TC by activating immune effector cells, leading to adaptive immune resistance [74, 75]. Additionally, trastuzumab modulates the function of TAMs, enhancing the co-expression of PD-L1 and the immunosuppressive molecule indoleamine 2,3-dioxygenase (IDO). This, in turn, weakens ADCP and promotes the formation of an immunosuppressive microenvironment [56]. CD47, as a “don’t eat me” signaling molecule, combines with PD-L1 to form a dual immune checkpoint network: CD47 inhibits phagocytosis by binding to signal regulatory protein alpha (SIRPα) on macrophages, while PD-L1 suppresses T-cell activity to limit adaptive immune responses. They cooperate and significantly impair anti-HER2 immunosurveillance [76, 77]. One study demonstrated that long noncoding RNAs exert their effects through miRNA sponge activity (adsorbing miR-141 and miR-340) and interaction with the transcription factor YBX1. This collectively promotes the transcriptional activation of PD-L1 and CD47, establishing an epigenetic regulatory network for immune evasion that contributes to trastuzumab resistance [78]. Preclinical studies have demonstrated that dual-specificity anti-PD-L1/SIRPα antibodies can simultaneously block both the PD-1/PD-L1 and CD47/SIRPα pathways, significantly enhancing antitumor immune responses and overcoming resistance [75, 79]. Moreover, data show that combining PD-1 inhibitors with trastuzumab in resistant patients can restore anti-HER2 immune activity and tumor-infiltrating lymphocyte levels may serve as a potential biomarker for treatment response [75]. These findings suggest that trastuzumab resistance arises from the remodeling of the immune microenvironment by TC via PD-L1- and CD47-mediated immune checkpoint networks. Targeting dual checkpoints may offer new strategies to overcome resistance (Fig. 2).

## Conclusions

In summary, trastuzumab resistance in HER2-positive GC is multifactorial, yet emerging evidence underscores the critical role of the TIME. The failure of agents that solely target HER2 signaling (e.g. lapatinib, pertuzumab) and the success of combinations with ICIs (e.g. pembrolizumab, ALX148) or anti-angiogenic agents highlight that modulating the TME is essential to overcome resistance. Future research may focus on identifying predictive biomarkers (such as TILs, PD-L1/CD47 expression) to select patients who will benefit from combined immunotherapy. Moreover, novel strategies that simultaneously target innate (e.g. CD47) and adaptive immune checkpoints, along with optimizing the sequencing of ADCs and immunotherapy, warrant further investigation. Ultimately, integrating TME-directed therapies with anti-HER2 agents holds promise for improving outcomes in this patient population.

## Authors’ contributions

H.L. and X.B.C. conceptualized the study. H.L. conducted the investigation and provided resources. H.L. and W.Z. wrote the original draft. W.Z. and S.Q.W. curated the data. W.Z. developed the methodology. S.Q.W. performed validation. S.Q.W., Y.H., and C.N. reviewed and edited the manuscript. Y.H. and C.N. performed formal analysis and visualization. X.B.C. acquired funding, administered the project, and supervised the study. All authors have read and agreed to the published version of the manuscript.
